# Supplementary material for: Context-dependent agricultural intensification pathways to increase rice production in India
Source: Nat Commun. 2024 Sep 27;15:8403. doi: 10.1038/s41467-024-52448-6 (PMC11436799; doi:10.1038/s41467-024-52448-6)
Supplement: Supplementary file 1 — Supplementary Information [file 41467_2024_52448_MOESM1_ESM.pdf]

Supplementary material to “Context-dependent agricultural intensification pathways to increase rice production in India”

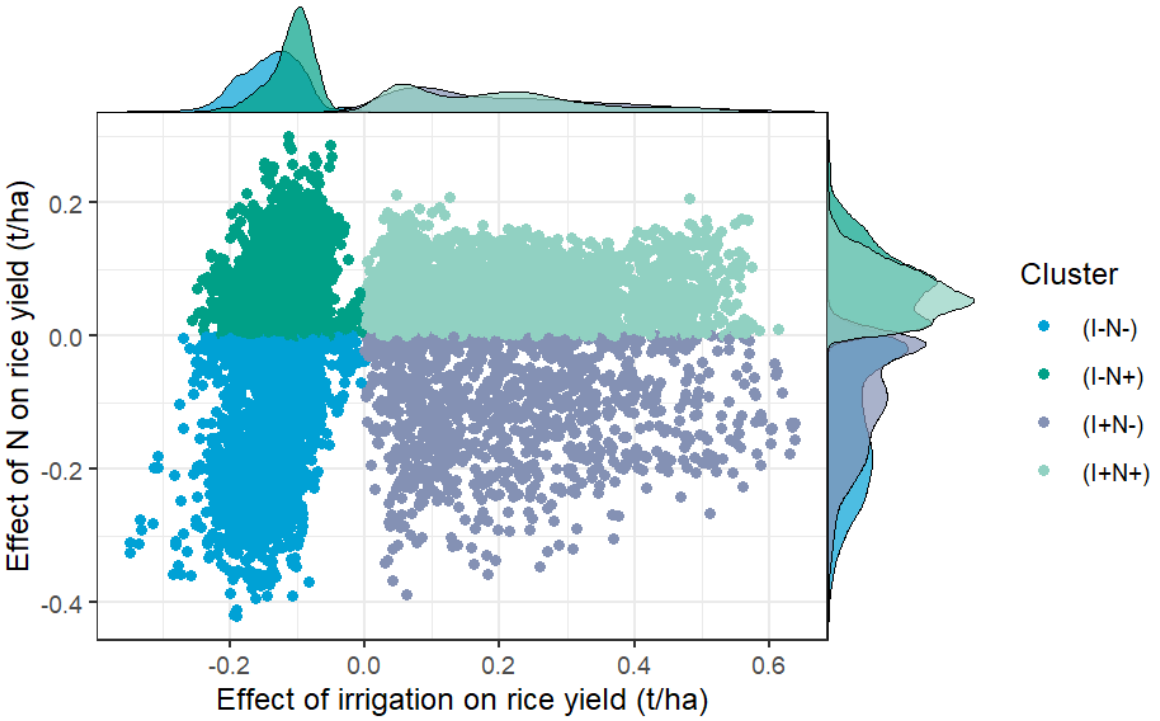

Supplementary Figure 1 | Clustering of surveyed fields into attainable yield gap ‘types’ in Eastern India based on the SHAP values for irrigation and N fertilizer, the two most important predictor variables for rice productivity in the region of Bihar and Easter Uttar Pradesh (n = 10,714).

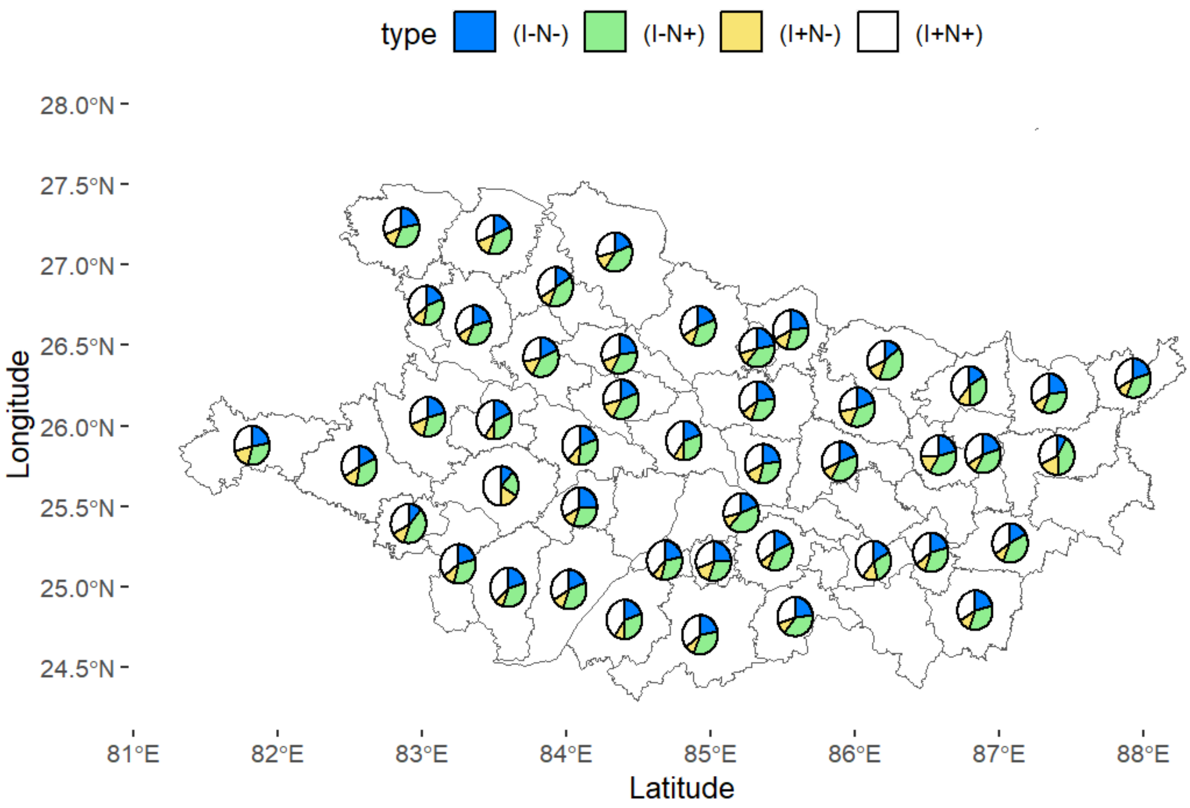

Supplementary Figure 2 | District-wise variation in yield gap cluster membership in Eastern India (n = 10,714).

Supplementary Table 1 | Change in rice production and input requirements per annum for targeted and 'blanket' management strategies based on *ex-ante* scenario analysis. For scenario descriptions, refer to *Methods* and Figure 5. Aggregated benefits are scaled to the rice production area of Eastern India as defined in this study (i.e., Bihar and adjacent districts in Eastern Uttar Pradesh)

	**Change in rice production (million tons)**	**Change in N (million tons)**	**Change in irrigation (million events)**
**Scenario 1:**<br>N blanket (125 kg N ha^{-1})	0.15	-0.016	N/A
**Scenario 2**:<br>N blanket (180 kg N ha^{-1})	0.57	0.22	N/A
**Scenario 3:**<br>N targeted	0.41	0.125	N/A
**Scenario 4:**<br>N and irrigation targeted	0.56	0.08	2.33

Note: The change in rice production and input use for N and irrigation are defined relative to the current farm practice. For Scenario 3, this implies an incremental NUE of 3.28 kg grain for every kg N applied above the current farm practice with an overall NUE of 22.7 kg grain kg^{-1} N applied, when accounting for grain output and N fertilizer input.

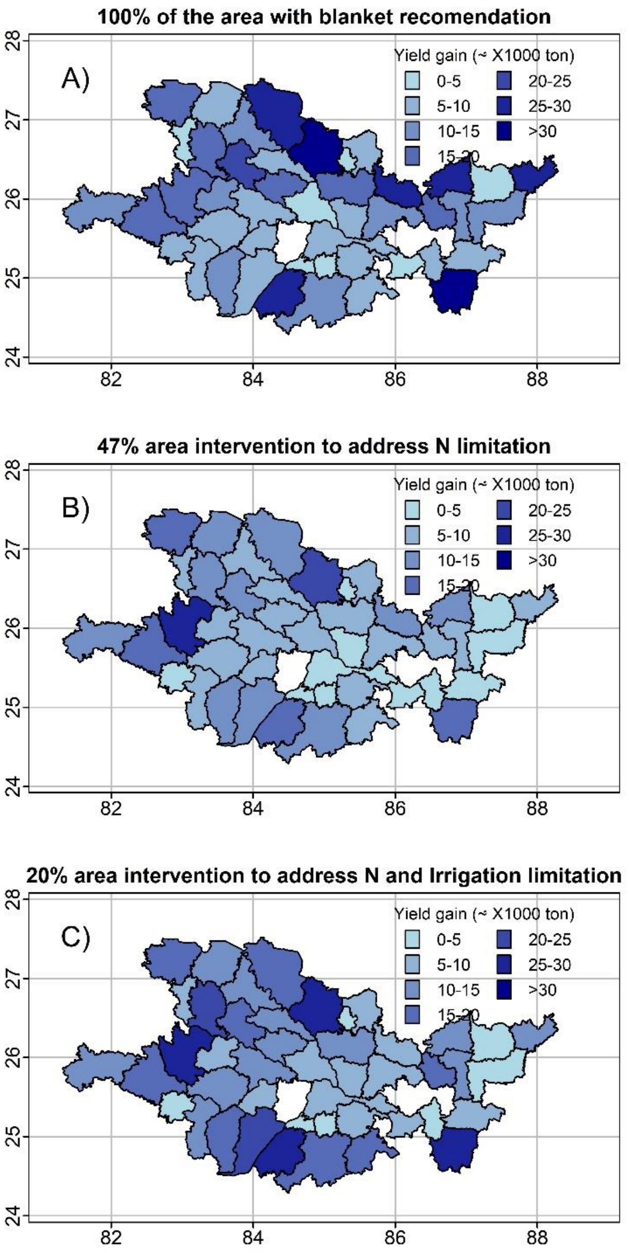

Supplementary Figure 3 | Additional rice yield in Bihar and Eastern Uttar Pradesh mapped at the district level under *ex ante* Scenarios 2 (A), 3 (B), and 4 (C). For a detailed description of the scenarios refer to *Methods* and Figure 5.

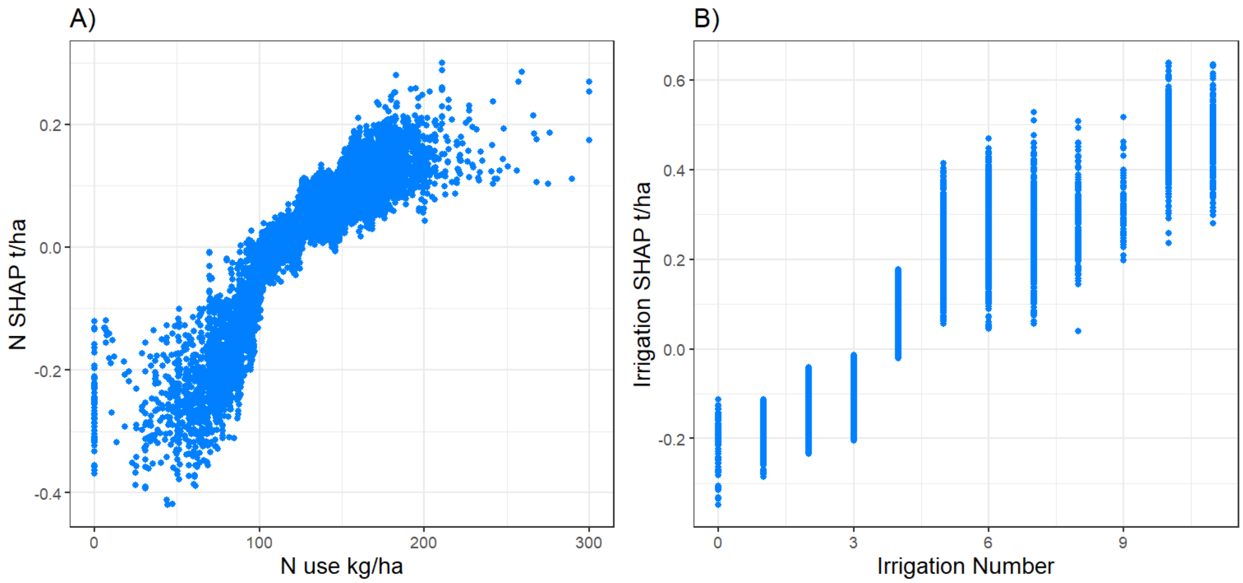

Supplementary Figure 4: SHAP dependence plot for the two most important management practices explaining rice yield variability in Eastern India A) Nitrogen use kg per ha, B) Irrigation number.

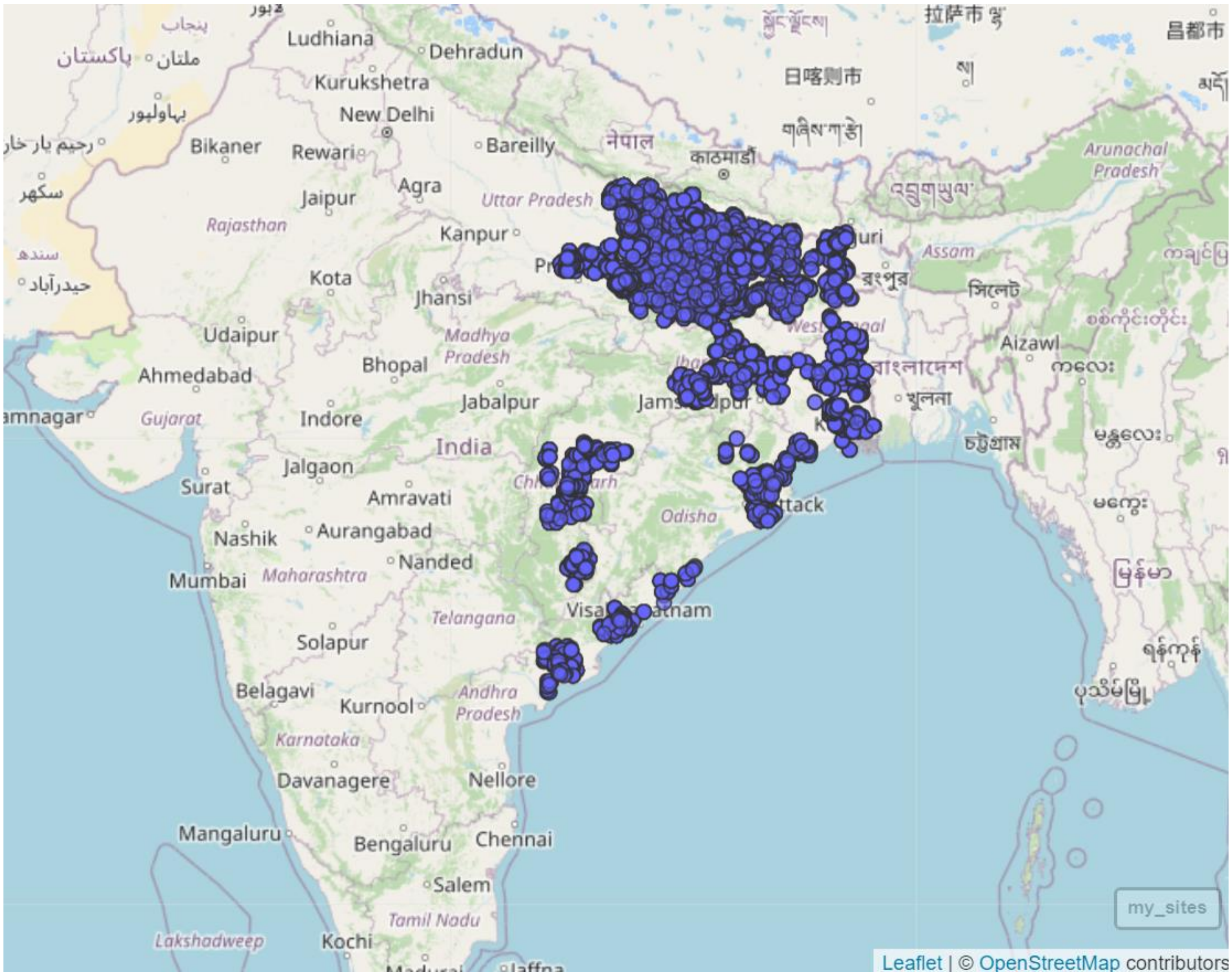

Supplementary Figure 5: The distribution of sampling point across seven states of Eastern India (n = 15,686).

Supplementary Table 2: Descriptive statistics of the variables used in yield modeling along with the yield for continuous variables.

Category	mean	sd	min	max
Yield (t/ha)	4.06	1.08	1.21	6.88
Total N applied (kg/ha)	127.07	35.99	0.00	300.00
Average maximum temperature	31.88	1.03	28.05	35.26
Average radiation	2032.98	220.08	1264.62	3000.00
Average minimum temperature	23.81	1.15	18.97	26.99
Number of irrigation	3.84	2.23	0.00	11.00
Average rainfall	652.54	227.37	103.61	1563.24
Total P applied (kg/ha)	47.60	24.30	0.00	191.67
Duration	125.01	13.32	87.00	185.00
Sowing date in Julian day	192.32	11.94	150.00	221.00
Total K applied (kg/ha)	11.73	16.77	0.00	108.80
