# Supplementary material for: Context-dependent agricultural intensification pathways to increase rice production in India
Source: Nat Commun. 2024 Sep 27;15:8403. doi: 10.1038/s41467-024-52448-6 (PMC11436799; doi:10.1038/s41467-024-52448-6)
Supplement: Supplementary file 3 — Description of Additional Supplementary Files [file 41467_2024_52448_MOESM3_ESM.pdf]

## Description of Additional Supplementary Files

File Name: Supplementary Data 1
Description: Named all_year_data.Rdata, contains all the raw data.

File Name: Supplementary Data 2
Description: Named Data_for_script_3.Rdata, contains the yield model and SHAPELY values, which was used for scenario and ex-ante analysis.

File Name: Supplementary Code 1
Description: This contains all the code script named from script_1 to script_4.
