# Supplementary material for: Context-dependent agricultural intensification pathways to increase rice production in India
Source: Nat Commun. 2024 Sep 27;15:8403. doi: 10.1038/s41467-024-52448-6 (PMC11436799; doi:10.1038/s41467-024-52448-6)
Supplement: Supplementary file 6 — Reporting Summary [file 41467_2024_52448_MOESM6_ESM.pdf]

nature portfolio

Corresponding author(s): Hari Sankar Nayak

Last updated by author(s): Aug 28, 2024

Reporting Summary

Nature Portfolio wishes to improve the reproducibility of the work that we publish. This form provides structure for consistency and transparency in reporting. For further information on Nature Portfolio policies, see our Editorial Policies and the Editorial Policy Checklist.

Statistics

For all statistical analyses, confirm that the following items are present in the figure legend, table legend, main text, or Methods section.

n/a	Confirmed	
☐	☒	The exact sample size (*n*) for each experimental group/condition, given as a discrete number and unit of measurement
☐	☒	A statement on whether measurements were taken from distinct samples or whether the same sample was measured repeatedly
☒	☐	The statistical test(s) used AND whether they are one- or two-sided<br>*Only common tests should be described solely by name; describe more complex techniques in the Methods section.*
☒	☐	A description of all covariates tested
☒	☐	A description of any assumptions or corrections, such as tests of normality and adjustment for multiple comparisons
☐	☒	A full description of the statistical parameters including central tendency (e.g. means) or other basic estimates (e.g. regression coefficient) AND variation (e.g. standard deviation) or associated estimates of uncertainty (e.g. confidence intervals)
☒	☐	For null hypothesis testing, the test statistic (e.g. *F*, *t*, *r*) with confidence intervals, effect sizes, degrees of freedom and *P* value noted<br>*Give P values as exact values whenever suitable.*
☒	☐	For Bayesian analysis, information on the choice of priors and Markov chain Monte Carlo settings
☒	☐	For hierarchical and complex designs, identification of the appropriate level for tests and full reporting of outcomes
☒	☐	Estimates of effect sizes (e.g. Cohen's *d*, Pearson's *r*), indicating how they were calculated

Our web collection on statistics for biologists contains articles on many of the points above.

Software and code

Policy information about availability of computer code

Data collection	The data was collected using Android based ODK tool. The detailed method is available in https://www.sciencedirect.com/science/article/pii/S2352340922008319. The data is attached with the submitted manuscript and also available online.
Data analysis	The R code is attached along with MS. All data analysis were conducted in R (4.2.3) with following package and version number, dplyr (1.1.4), caret (6.0.93), range (0.14.1), iml (0.11.1), geodata (0.5.3), terra (1.7.55), tidyverse (1.3.2), ggpubr (0.6.0) and dependencies, data.table (1.14.2), and gridExtra (2.3). The hotspot analysis was carried out in ArcGIS Pro v.2.9.0.

For manuscripts utilizing custom algorithms or software that are central to the research but not yet described in published literature, software must be made available to editors and reviewers. We strongly encourage code deposition in a community repository (e.g. GitHub). See the Nature Portfolio guidelines for submitting code & software for further information.

Data

Policy information about availability of data

All manuscripts must include a data availability statement. This statement should provide the following information, where applicable:

- Accession codes, unique identifiers, or web links for publicly available datasets
- A description of any restrictions on data availability
- For clinical datasets or third party data, please ensure that the statement adheres to our policy

Provide your data availability statement here.

Research involving human participants, their data, or biological material

Policy information about studies with human participants or human data. See also policy information about sex, gender (identity/presentation), and sexual orientation and race, ethnicity and racism.

Reporting on sex and gender	Random sample collected irrespective of sex or gender of participating farmer respondents.
Reporting on race, ethnicity, or other socially relevant groupings	NA
Population characteristics	NA
Recruitment	NA
Ethics oversight	The research conducted herein was reviewed by and complies with standards established by the Research Ethics Committee of the International Maize and Wheat Improvement Center (CIMMYT) as described in policy number DDG-POL-04–2019. The ethics review code for this study is IREC.2019.06. Verbal consent was obtained from all survey participants.

Note that full information on the approval of the study protocol must also be provided in the manuscript.

Field-specific reporting

Please select the one below that is the best fit for your research. If you are not sure, read the appropriate sections before making your selection.

☐ Life sciences ☐ Behavioural & social sciences ☒ Ecological, evolutionary & environmental sciences

For a reference copy of the document with all sections, see nature.com/documents/nr-reporting-summary-flat.pdf

Ecological, evolutionary & environmental sciences study design

All studies must disclose on these points even when the disclosure is negative.

Study description	Data on agronomic management practices were collected with ODK-based survey, which was supplemented with bio-physical variables (gridded climate and soil data) to develop a complete data stack. A machine learning yield model was built which was interpreted with local explanation techniques; thereafter, ex-ante scenario and spatial analysis was used to characterize where agronomic practice changes are anticipated to bring the greatest benefits across performance indicators.
Research sample	The total number of farms include 15,876 field from seven Indian states: Uttar Pradesh, Bihar, Jharkhand, West Bengal, Andhra Pradesh, Chattisgarh, and Odisha.
Sampling strategy	Districts are the principal survey units. From each district, villages were selected with a random draw using probability-proportionate-to-population size method. Extremely small and large villages including all urban locations were excluded. From each village, seven households were selected randomly from electoral rolls with additional household selected if potential participants refused to participate or didn't grow rice.
Data collection	Data was collected through computer-assisted personal interview of farmers using Open Data Kit (ODK). The digital survey form was available on mobile phones of trained enumerators and was designed to minimize data entry errors. Each survey captured variables around rice production practices of farmers' largest plot starting with land preparation, establishment method, crop variety and planting time through to crop yield. Detailed modules captured information on fertilizer application, irrigation, weed management, biotic and abiotic stresses. Completed surveys were sent by enumerators to a cloud-based server of ODK called Aggregate. Auto-compiled raw dataset was downloaded from server.
Timing and spatial scale	Data were collected every year beginning from the 2017 rainy season, 2018 rainy season, and 2019 rainy season from seven rice producing Indian states.
Data exclusions	The survey questionnaire was designed to flag the unrealistic values and to prompt enumerators to address potential errors by re-asking relevant questions. For modeling purposes, yield values less than 1.2 ton per hectare and more than 6.9 ton per ha were excluded from the Eastern India case study region (n=101 fields). The threshold were chosen based on data distribution from the box plot interquartile range. Based on same logic, 10, 26, 19, and 34 data points were excluded from Jharkhand, Odisha, West Bengal, and Chattisgarh, respectively. After all exclusions, total sample size is 15,686.
Reproducibility	We have provided the data and script, which can be used to generate the output. We have provided the exact model and SHAP values which was used for ex-ante scenario analysis.
Randomization	As this is an observational and modeling study, no treatments or randomization was implemented.
Blinding	NA

Did the study involve field work? ☐ Yes ☒ No

Reporting for specific materials, systems and methods

We require information from authors about some types of materials, experimental systems and methods used in many studies. Here, indicate whether each material, system or method listed is relevant to your study. If you are not sure if a list item applies to your research, read the appropriate section before selecting a response.

Materials & experimental systems

n/a	Involved in the study
☒	☐ Antibodies
☒	☐ Eukaryotic cell lines
☒	☐ Palaeontology and archaeology
☒	☐ Animals and other organisms
☒	☐ Clinical data
☒	☐ Dual use research of concern
☒	☐ Plants

Methods

n/a	Involved in the study
☒	☐ ChIP-seq
☒	☐ Flow cytometry
☒	☐ MRI-based neuroimaging

Plants

Seed stocks	NA
Novel plant genotypes	NA
Authentication	NA
